# Xanthine calculi in a patient with Lesch-Nyhan syndrome and factor V Leiden treated with allopurinol: case report

**DOI:** 10.1186/s12887-018-1197-5

**Published:** 2018-07-12

**Authors:** Lisa B. E. Shields, Dennis S. Peppas, Eran Rosenberg

**Affiliations:** 1 Norton Neuroscience Institute, Norton Healthcare, Louisville, KY 40202 USA; 2 Norton Children’s Urology, Norton Healthcare, Louisville, KY 40207 USA

**Keywords:** Pediatrics, Lesch-Nyhan, Kidney stones, Allopurinol, Factor V Leiden

## Abstract

**Background:**

Lesch-Nyhan syndrome is a rare inborn error of purine metabolism marked by a complete deficiency of the enzyme hypoxanthine-guanine phosphoribosyltransferase (HPRT). Inherited as an X-linked recessive genetic disorder that primarily affects males, patients with Lesch-Nyhan syndrome exhibit severe neurological impairments, including choreoathetosis, ballismus, cognitive dysfunction, and self-injurious behavior. Uric acid levels are usually abnormally high, leading to kidney and bladder stones which often necessitate urological intervention. Factor V Leiden is an autosomal dominant disorder of blood clotting associated with hypercoagulability, thrombophilia, and renal disease.

**Case presentation:**

We present the first reported case of xanthine calculi in a patient with Lesch-Nyhan syndrome and Factor V Leiden who was treated with allopurinol. A renal ultrasound and CT scan demonstrated bilateral staghorn calculi in the kidneys as well as nephrocalcinosis. Two years earlier the patient underwent cystoscopy with bilateral ureteroscopy and laser lithotripsy, and he was stone free afterwards. The patient subsequently underwent bilateral percutaneous nephrolithotomy (PCNL) and was stone free following the procedure. Patients with endogenous overproduction of uric acid who are being treated with allopurinol have a higher chance of developing xanthine stones.

**Conclusions:**

Pediatricians treating these children should be aware of these rare conditions and promptly manage the potential complications that may require medical or surgical intervention.

## Background

Initially described in 1964, Lesch-Nyhan syndrome is characterized by a triad of hyperuricemia, central nervous system dysfunction, and familial inheritance and has an incidence of 1:100,000 to 1:300,000 [1, 2]. Neurological abnormalities include spasticity, cognitive impairment, impulsivity, hematological disorders such as megaloblastic anemia or microcytic anemia, and compulsive self-injurious behavior, specifically, lip biting or finger chewing [1, 3–5]. Uric acid overproduction often leads to lithiasis and gout [5].

The enzyme hypoxanthine-guanine phosphoribosyltransferase (HPRT) plays an important role in uric acid synthesis and purine metabolism [6]. HPRT-deficient patients have purine overproduction and elevated uric acid levels. A spectrum of diseases results from mutations of the HPRT gene, and the severity of the molecular defect correlates with the clinical phenotype [3, 5, 6]. Partial HPRT deficiency as observed in Kelley-Seegmiller syndrome is manifested by excessive purine synthesis, gout, and no neurological involvement [7, 8]. Inherited as an X-linked recessive disorder generally affecting males, Lesch-Nyhan syndrome represents the most severe phenotype [5].

Factor V Leiden, the most common cause of inherited thrombophilia, is associated with a mutation making it resistant to the action of the natural anticoagulant activated protein C (APC) [9]. APC is unable to prevent Factor V Leiden from producing more fibrin. Heterozygous Factor V Leiden is found in approximately 5% of the Caucasian population and poses an increased risk of developing deep venous thrombosis, pulmonary embolism, and renal disease [9–13]. Elevated homocysteine levels are associated with an increased risk for atherosclerosis and venous thrombosis as well as microalbuminuria, renal dysfunction, and megaloblastic anemia [14, 15].

We report the first case of xanthine calculi in a patient with Lesch-Nyhan syndrome and Factor V Leiden who was treated with allopurinol and underwent several urological procedures to remove calculi. We discuss the pathophysiology of xanthine stones, the use of allopurinol in patients with HPRT deficiency experiencing uric acid overproduction, the consequences of allopurinol overdosing, the challenges associated with the combined diagnoses of Lesch-Nyhan syndrome and Factor V Leiden, and the myriad medical and surgical managements for urolithiasis associated with Lesch-Nyhan syndrome.

## Case presentation

### Case report

A 12-year-old boy (height: 51 in.; weight; 44 lbs. 1.5 oz. [20.0 kg]; BMI: 11.93 kg/m^2^) with a history of Lesch-Nyhan syndrome presented to our office with a 1 ½ month history of dysuria, hematuria, and pain secondary to nephrolithiasis. He suffered from a non-verbal learning disorder associated with a developmental delay, was wheelchair-dependent, and had undergone extraction of 10 teeth due to biting and grinding his teeth. Due to his self-mutilating behavior, he wore braces on his arms and had surgery of his left thumb as a result of biting himself. Two years prior to presentation, the patient underwent a cystoscopy with bilateral ureteroscopy due to xanthine stones. He was stone free following the procedure. The patient’s mother denied a family history of kidney stones, thromboembolism and gout. At the age of 18 months, the boy underwent a test for organic acids in his urine which revealed highly elevated hypoxanthine without an elevation of xanthine and with a slight elevation of uracil. He was diagnosed clinically with Lesch-Nyhan syndrome at that time based on a triad of uric acid overproduction, neurologic dysfunction, and cognitive and behavioral disturbances.

Uric acid crystals were noted intermittently in the patient’s diaper which had increased significantly in the preceding days. He had been treated with the medication allopurinol since he was 2 years old. At the time of presentation, the dose of allopurinol was 200 mg administered once per day. He had never experienced gout. The patient was prescribed potassium citrate.

### Diagnostic tests

**Fig. 1:**
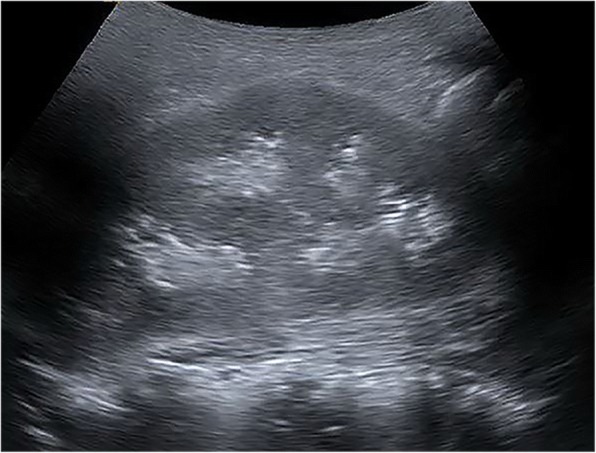
Renal ultrasound demonstrated multiple calculi and nephrocalcinosis of the kidneys bilaterally

A renal ultrasound demonstrated multiple calculi in both kidneys with the largest measuring 1.8 cm in the right kidney as well as echogenic material in the medullary pyramids bilaterally suggesting nephrocalcinosis (Fig. 1). There were no masses, hydronephrosis, or hydroureter. A urinalysis revealed the following: specific gravity = 1.010; pH = 7.0; leukocyte esterase = 500; trace microscopic hematuria; and negative nitrite. The urine culture was negative. The uric acid level in blood was 2.9 mg/dL (Normal range: 2.5–8.5 mg/dL). Uric acid levels in blood collected in the previous 2 years were all in the normal range, specifically, 5.0 mg/dL, 2.6 mg/dL, and 3.2 mg/dL. There was no evidence of renal failure. The patient underwent six separate urinalyses between the ages of 9 and 12, all of which demonstrated a pH of either 6 or 7.

A CT scan of the abdomen and pelvis demonstrated staghorn calculi in both kidneys, involving all calyces.

### Surgery

The patient underwent a right percutaneous nephrolithotomy (PCNL) 2 months after presentation. During the hospital stay, the patient was noted to be anemic and received 5 days of epogen and iron supplementation. He developed a thrombus in the right cephalic vein and was diagnosed with heterozygous Factor V Leiden with elevated homocysteine (16.2 umol/L [Normal range: 6.6–14.8 umol/L]). The methylene tetrahydrofolate reductase (MTHFR) and prothrombin genes were negative. Folic acid was initiated. The Factor V Leiden mutation was detected through a polymerase chain reaction (PCR) test that utilized microarray-based oligonucleotide hybridization and signal amplification to detect the Factor V Leiden mutation.

**Fig. 2:**
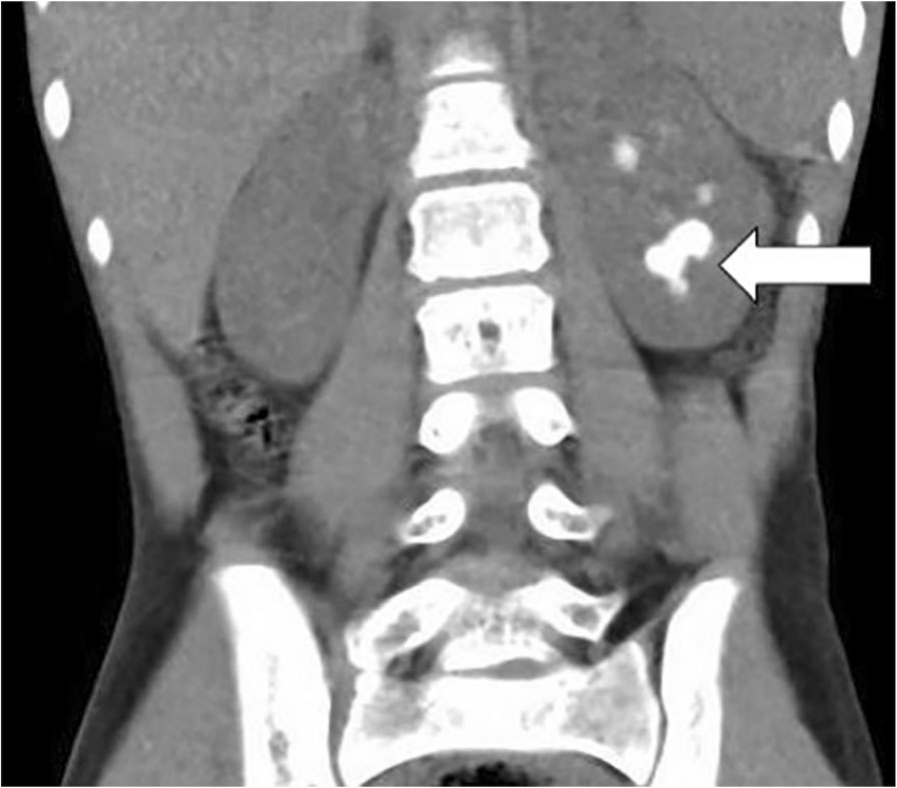
CT scan of the abdomen and pelvis showed staghorn calculi in the left kidney

Seven weeks later, the patient began experiencing aspiration of liquids, gastroesophageal reflux, and markedly delayed gastric emptying, necessitating a laparoscopic Nissen fundoplication with gastrostomy tube and open pyloroplasty. The patient continued to suffer from nephrolithiasis, with visualization of a left renal calculus greater than 2 cm. A CT scan demonstrated staghorn calculi in the left kidney (Fig. 2). He underwent a left PCNL and left nephrostomy tube exchange 6 months after the right-sided procedure. A nephrogram revealed evidence of a filling defect in the lower pole calyx which may represent organizing blood clot or residual stone. Renal ultrasounds performed 6 and 9 months later demonstrated extensive bilateral nephrocalcinosis without hydronephrosis or hydroureter. There was no evidence of calculi in the collecting systems.

The calculi that were removed during the ureteroscopies, the ones that the patient had passed while seen as an outpatient, and those that were sent for analysis following the PCNL were all consistent with xanthine calculi.

## Discussion

A classic feature of Lesch-Nyhan syndrome is hyperuricemia due to a metabolic deficit caused by a HPRT mutation [16]. The HPRT defect results in the accumulation of hypoxanthine which is oxidized to xanthine by xanthine oxidase and further oxidized to uric acid [5, 17]. Hyperuricemia may lead to hyperuricosuria, uric acid stone formation, and uric acid crystal nephropathy [16]. If the hyperuricemia remains untreated, conditions such as gouty arthritis and fatal uric acid nephropathy may arise [5, 17].

Allopurinol, an inhibitor of xanthine oxidase, stimulates xanthine and hypoxanthine urinary excretion and decreases uric acid production [2, 18]. In contrast with hypoxanthine, the solubility of xanthine in urine is low. While allopurinol is often beneficial in the treatment of the hyperuricemic state associated with Lesch-Nyhan syndrome, there is a risk of developing xanthine stones when the urine is saturated with xanthine [18]. Torres and colleagues performed a 22-year investigation of the use of allopurinol in patients with Lesch-Nyhan syndrome and partial HPRT deficiency [19, 20]. They reported a 47% mean reduction of serum urate concentration, a mean 74% reduction in urinary uric acid-to-creatinine ratio, and increased hypoxanthine and xanthine urinary excretion rates by 5.4 and 9.5 times, respectively [20]. These authors concluded that allopurinol was a safe and efficacious drug for the treatment of uric acid overproduction and did not influence the neurological features associated with Lesch-Nyhan syndrome [19, 20].

Pediatric urinary lithiasis is rarely encountered and, when observed, consists often of calcium oxalate stones [17, 18, 21]. The most common cause of xanthine calculi is primary hereditary xanthinuria which is an autosomal recessive disorder due to a deficiency of the enzyme xanthine oxidase [17, 22]. Individuals with Lesch-Nyhan syndrome are at risk of developing xanthine calculi as a result of treatment with allopurinol [16], however, a paucity of cases have been reported [17–19, 23]. The mechanism of xanthine stone formation may be related to the significant increase of urinary oxypurinol, the metabolite of allopurinol, in response to allopurinol treatment [22]. Patients may present with renal failure secondary to obstructive uropathy and are subsequently diagnosed with Lesch-Nyhan syndrome [16, 24] or may suffer from Lesch-Nyhan syndrome and experience renal failure secondary to uric acid nephropathy or stone obstruction [2, 18].

A fine line exists between lowering serum urate sufficiently to prevent the painful manifestations of gout while not producing excessive xanthine in the urine. Cameron and colleagues stressed the importance of careful monitoring of allopurinol especially in patients with Lesch-Nyhan syndrome as the total urinary oxypurine excretion is sensitive to allopurinol which may result in xanthine and oxypurinol calculi [25]. The allopurinol dose should be closely monitored and reduced to no more than 5 mg/kg per 24 h in children or 100 mg/24 h in adults [25]. The boy presented in our case was treated with an allopurinol dose (10 mg/kg) that was so excessive that his serum urate was at the lower end of normal (5.0 mg/dL, 2.6 mg/dL, and 3.2 mg/dL). Thus, his large urate production was diverted into xanthine and hypoxanthine. Furthermore, Torres and colleagues suggested maintaining the urinary hypoxanthine excretion rate higher than that of xanthine and keeping the serum urate concentration between 5.0 mg/dL and 7.0 mg/dL to prevent xanthine lithiasis associated with allopurinol use in HPRT deficiency [20]. However, the boy in our case had a serum urate level far below this range.

The dangers of excessive allopurinol in this report serves as a significant educational message for physicians. Our report represents a situation of allopurinol overdosing. First, Torres and colleagues’ recommendation of keeping the serum urate concentration between 5.0 mg/dL and 7.0 mg/dL was not followed [20]. Secondly, the dose of allopurinol was double the recommended dose of 5 mg/kg [25]. The boy developed xanthine lithiasis as a consequence of these factors, placing him at risk of developing renal failure.

We report the first case in the literature of a patient with Lesch-Nyhan syndrome and Factor V Leiden who was treated with allopurinol and subsequently developed xanthine calculi. Bilateral staghorn calculi were noted, involving all calyces. Nephrocalcinosis was also observed in the medullary pyramids bilaterally. The uric acid levels in the blood remained in the normal range throughout the course of urological follow-up.

Our patient’s combined diagnoses of Lesch-Nyhan syndrome and Factor V Leiden created several challenges. The urological course of treating the xanthine calculi was complicated by his Factor V Leiden, with the development of a thrombus in the right cephalic vein. Due to the risk of thrombophilia associated with Factor V Leiden, afflicted individuals are encouraged to remain active and refrain from being immobile for long periods of time. The boy in our report with Lesch-Nyhan syndrome had motor dysfunction and required a wheelchair, preventing him from the recommended walking. Our patient also had anemia and an increased homocysteine level which may be associated with microalbuminuria, megaloblastic anemia, and renal dysfunction. Similarly, patients with Lesch-Nyhan syndrome may experience hematological disorders such as megaloblastic anemia or microcytic anemia in addition to their uric acid overproduction. The renal and hematological risk factors associated with Lesch-Nyhan syndrome, Factor V Leiden, and elevated homocysteine levels may have exacerbated this boy’s condition. We believe it is a coincidence that the boy was diagnosed with both heterozygous Lesch-Nyhan syndrome and Factor V Leiden. Lesch-Nyhan syndrome is caused by a deficiency of the enzyme HGPRT due to a mutation in the HPRT gene located on the X chromosome, whereas Factor V Leiden (rs6025) is an autosomal disorder marked by a mutation of human factor V located on chromosome 1q24.2. We have not discerned a genetic correlation between these two conditions.

The first-line treatment of uric acid stones or uric acid crystal nephropathy in individuals with Lesch-Nyhan syndrome consists of medical management (Table 1) [2, 16–18, 22, 23]. It has been suggested that raising the dose of allopurinol may be successful in the decreasing the frequency of stone formation by increasing the hypoxanthine to xanthine ratio [26]. Contrarily, urinary alkalization coupled with reducing the dose of allopurinol may prove more efficacious in preventing xanthine stone formation [17]. If medical management fails or is not tolerated, invasive procedures may be performed (Table 1) [16].

**Table 1:** Treatment of Urolithiasis Associated with Lesch-Nyhan syndrome

Medical Management	Invasive Procedures
ᅟ■ High fluid intake ᅟ■ Maintain alkaline urine with potassium or sodium citrate to prevent uric acid stone formation (urinary pH: 6.5–7.0) ᅟ■ Gradually increase allopurinol dose from 2.5 mg/kg doses per day to 10 mg/kg per day and administer 2–3 doses per day ᅟ■ Monitor purine metabolites of allopurinol in blood and urine ᅟ■ Assess urinary uric acid, xanthine, hypoxanthine, and oxypurinal excretion to determine accurate allopurinol dosage ᅟ■ Avoid excess dietary purines, calcium, salts ᅟ■ Routine renal ultrasound to monitor for uric acid nephropathy	■ Percutaneous nephrolithotomy ■ Shock wave lithotripsy ■ Ureteroscopy ■ Open surgery

Despite proper medical management consisting of aggressive hydration, an appropriate dose of the allopurinol which was not adjusted during treatment, use of potassium citrate, and routine renal ultrasounds, the patient presented herein underwent numerous urological procedures for calculi, including a cystoscopy with bilateral uteroscopy and laser lithotripsy with bilateral placement of ureteral stents followed by bilateral PCNLs 2 years later. Renal ultrasounds performed 6 and 9 months after the urological interventions showed extensive bilateral nephrocalcinosis without hydronephrosis, hydroureter, or calculi, indicating that the medical and surgical management of the xanthine calculi had proven successful.

## Conclusions

Pediatricians should be aware of the rare phenomena of Lesch-Nyhan syndrome and Factor V Leiden and the potential renal disorders inherent in both conditions. To our knowledge, this is the first case involving a single patient who was diagnosed with the genetic diseases Lesch-Nyhan syndrome and Factor V Leiden and experienced xanthine calculi after consuming allopurinol. Prompt medical management of xanthine calculi by a high fluid intake, maintaining an alkaline urine, and monitoring for uric acid nephropathy is warranted. Furthermore, awareness of the consequences of allopurinol overdosing is imperative in the management of patients with Lesch-Nyhan syndrome to decrease the likelihood of renal failure.

## Abbreviations

APC: Activated protein C
HPRT: Hypoxanthine-guanine phosphoribosyltransferase
MTHFR: Methylene tetrahydrofolate reductase
PCNL: Percutaneous nephrolithotomy
